# Investigating Bergamot Essential Oil (BEO) Properties: Cytoprotection in Neuronal Cells Exposed to Heavy Metals and Antibacterial Activities

**DOI:** 10.3390/antiox14040400

**Published:** 2025-03-27

**Authors:** Alexia Barbarossa, Rosanna Mallamaci, Eleonora Spinozzi, Filippo Maggi, Maria Noemi Sgobba, Antonio Rosato, Alessia Carocci, Daniela Meleleo

**Affiliations:** 1 Department of Pharmacy–Pharmaceutical Sciences, University of Bari “Aldo Moro”, 70125 Bari, Italy; alexia.barbarossa@uniba.it (A.B.); antonio.rosato@uniba.it (A.R.); 2 Department of Biosciences, Biotechnologies and Environment, University of Bari “Aldo Moro”, 70125 Bari, Italy; rosanna.mallamaci@uniba.it (R.M.); maria.sgobba@uniba.it (M.N.S.); 3 Chemistry Interdisciplinary Project (ChIP) Research Center, School of Pharmacy, University of Camerino, 62032 Camerino, Italy; eleonora.spinozzi@unicam.it (E.S.); filippo.maggi@unicam.it (F.M.); 4 Department of Science of Agriculture, Food, Natural Resources and Engineering, University of Foggia, 71122 Foggia, Italy; daniela.meleleo@unifg.it

**Keywords:** heavy metals, neurotoxicity, oxidative stress, SH-SY5Y, *Citrus bergamia* essential oil, antibacterial, Alzheimer’s disease

## Abstract

Bergamot [*Citrus* × *limon* (L.) Osbeck, syn. *C.* × *bergamia* (Risso) Risso & Poit.] is primarily cultivated in the Calabria region of Italy and exploited in the food and perfumery industry. The epicarp of its fruit is a rich source of essential oil (BEO) containing mainly monoterpenes, which are known for their diverse biological activities, including antimicrobial, anti-inflammatory, antiproliferative, and neuromodulatory effects. Emerging evidence suggests that oxidative stress plays a central role in the pathogenesis of neurodegenerative diseases, particularly Alzheimer’s disease (AD), where it contributes to neuronal dysfunction and cell death. Moreover, heavy metal exposure has been identified as a key environmental factor exacerbating oxidative stress and neurodegeneration in AD. This study aimed to explore whether BEO could mitigate heavy metal (Cd^2+^, Hg^2+^, and Pb^2+^)-induced neurotoxicity in SH-SY5Y cells, a model system for brain cells. MTT and calcein-AM assays were performed to examine the viability of the SH-SY5Y cells after exposure to each heavy metal itself, or in combination with BEO, whereas the LDH assay was carried out to determine the effects of BEO towards necrotic cell death induced by heavy metals. Furthermore, DCFH-DA was performed to determine whether BEO could protect SH-SY5Y from heavy metal-induced oxidative stress. This study also investigated the antibacterial properties of BEO on different Gram-positive and Gram-negative bacterial strains belonging to the ATCC collection. These results suggest that BEO may help counteract heavy metal-induced neuronal damage, particularly Cd^2+^ toxicity, potentially reducing one of the environmental risk factors associated with AD. Additionally, its antimicrobial properties reinforce its relevance in preventing infections that may contribute to neuroinflammation in AD.

## 1. Introduction

Bergamot essential oil (BEO) is obtained by cold pressing the epicarp and mesocarp of *Citrus* × *limon* (syn. *C.* × *bergamia*), a hybrid citrus primarily cultivated in Calabria, Italy, where it holds the Protected Designation of Origin (PDO) status [1,2]. Rich in bioactive terpenes, BEO is widely used in the perfume, cosmetic, pharmaceutical, and food industries due to its unique sensory properties and pharmacological activities including its ability to impact the cardiovascular system, [3] its antimicrobial effects against common pathogens, [4] and its neuropharmacological activities [5].

Beyond its general health benefits, BEO has been shown to modulate neurotransmitter function in the hippocampus, affecting synaptic plasticity and neuronal survival. Studies indicate its effect in pain modulation [6], as well as its ability to reduce cell death induced by NMDA glutamate receptor activation [7]. Its primary constituents—limonene, γ-terpinene, β-pinene, linalool, and linalyl acetate, which account for over 90% of its composition, contribute to its antioxidant capacity by scavenging free radicals and preventing oxidative damage [8]. Additionally, limonene contributes to the stabilization of cellular redox homeostasis by directly interacting with free radicals, thereby preventing lipid peroxidation and biomolecules oxidative damage [8].

The increasing environmental exposure to heavy metals such as cadmium (Cd^2+^), mercury (Hg^2+^), and lead (Pb^2+^) has raised significant concerns due to their well-documented neurotoxic effects. These metals are implicated in various neurological disorders, including cognitive decline and neuronal dysfunction, which are key features of neurodegenerative diseases, such as Alzheimer’s disease (AD) [9]. Indeed, they have been reported to promote aggregation of amyloid beta (Aβ) peptides and tau phosphorylation, leading to the formation of amyloid plaques and neurofibrillary tangles, hallmark features of AD pathology. Furthermore, they induce epigenetic changes, such as DNA methylation and histone modification, which can influence gene expression related to AD and contribute to its progression [10,11,12]. Moreover, these metals, by disrupting redox homeostasis and impairing mitochondrial function, promote oxidative stress, a crucial factor in AD pathogenesis, ultimately increasing neuronal susceptibility to degeneration. Heavy metals further contribute to oxidative stress by increasing ROS production and depleting antioxidant defenses. Particularly, Cd^2+^, Hg^2+^, and Pb^2+^ deplete glutathione (GSH) and deactivate critical antioxidant enzymes, exacerbating intracellular oxidative stress and leading to neuronal cell death [13,14,15,16,17].

Furthermore, growing evidence suggests that exposure to heavy metals also adversely affects the immune system, leading to an impaired immune response and increased susceptibility to infections. The compromised immunity may exacerbate the overall health impact of heavy metal exposure, promoting chronic infections [18]. In addition, the hypothesis that microbial infections could be a causative factor in AD is gaining traction. Pathogens may directly infect the brain or indirectly cause neuroinflammation, potentially triggering AD pathology [19]. Therefore, there is a growing interest in identifying natural compounds capable of mitigating the toxic effects of heavy metals on neural tissue, while providing prevention against infections. Such compounds should not only counteract the oxidative stress and neuronal damage induced by heavy metals, while reducing susceptibility to infections, which are often exacerbated by the immunosuppressive effects of metals exposure. Among the natural products, various essential oils have been identified to possess neuroprotective properties that may be beneficial in managing neurodegenerative diseases. In addition to their neuroprotective effects, some essential oils have also demonstrated the ability to mitigate heavy metal-induced toxicity by reducing oxidative stress, modulating inflammatory responses, and enhancing cellular defense mechanisms [20]. For example, several in vitro and in vivo studies demonstrated that *Lavandula angustifolia* essential oil, rich in linalool and linalyl acetate, is able to reduce oxidative stress, modulate the cholinergic system, and affect the GABAergic system, thus reducing neuronal excitability [21]. In addition to its neuroprotective effects, lavender essential oil also exhibits significant antimicrobial properties. Studies have shown that it possesses broad-spectrum antibacterial activity against both Gram-positive and Gram-negative bacteria, including *Staphylococcus aureus* and *Klebsiella pneumoniae*. Its antimicrobial efficacy is primarily attributed to linalool and linalyl acetate, which can disrupt bacterial cell membranes, inhibit biofilm formation, and interfere with microbial enzymatic activity [22]. Similarly, sweet orange essential oil, which contains both limonene and linalool, has been studied for its potential to reduce oxidative stress and protect neuronal cells from damage due to its ability to inhibit neuroinflammation and modulate neurotransmitter system [23]. In this context, BEO has been shown not only to counteract oxidative stress but also to enhance immune defenses [24,25]. Furthermore, recent studies have provided further insights into the neuroprotective potential of BEO components in the context of neurodegenerative diseases. Linalool has been demonstrated to attenuate Aβ42-induced oxidative stress and mitochondrial dysfunction in neuronal cells, thereby preventing apoptosis and improving cell survival [26]. Additionally, limonene has been shown to reduce Aβ aggregation and protect against Aβ-induced toxicity by modulating oxidative stress and inflammatory pathways [27]. These findings highlight the potential role of BEO in mitigating key pathological processes in AD, reinforcing its value as a neuroprotective agent. In addition to these effects, it exhibits significant antibacterial activity against both Gram-positive and Gram-negative bacteria, including multi-drug-resistant strains of clinical interest [28]. However, despite the increasing body of evidence supporting the several activities of BEO, including its antioxidant and neuroprotective potential, the specific actions of BEO in protecting neuronal cells from the toxic effects of heavy metals such as Cd^2+^, Hg^2+^, and Pb^2+^ remain underexplored.

This study aims to investigate the cytoprotective effects of BEO in neuronal SH-SY5Y cells exposed to Cd^2+^, Hg^2+^, and Pb^2+^, focusing on its ability to attenuate metal-induced toxicity and oxidative stress. We seek to determine the potential of BEO as a therapeutic candidate for mitigating heavy metal-induced neurotoxicity. Additionally, considering the rising concerns about antibiotic resistance, we also evaluated the antibacterial activity of BEO against a range of Gram-positive and Gram-negative bacterial strains, expanding the scope of its pharmacological profile. Together, these findings may contribute to a deeper understanding of the therapeutic potential of BEO in both neuroprotection and antimicrobial defense.

## 2. Materials and Methods

### 2.1. Chemicals

BEO (L.2309L) was obtained by cold pressing (rectified) and purchased from NASOTERAPIA (Padova, Italy). The following chemicals were purchased from the Sigma-Aldrich S.p.a. (Milan, Italy): [3-(4,5-dimethylthiazol-2-yl)-2,5-diphenyl-tetrazolium bromide] (MTT), nicotinamide adenine dinucleotide phosphate (NADPH), ethylenediaminetetraacetic acid sodium salt (Na-EDTA), sodium pyruvate, hydrogen peroxide, CdCl_2_, HgCl_2_, Pb(NO_3_)_2_, L-glutamine, trypsin, LDH, and 2′,7′-dichlorofluorescein diacetate. The following cell culture materials were purchased: high-glucose (4.5 g L^−1^) Dulbecco’s modified Eagle medium (DMEM) and fetal bovine serum (FBS, PAN Biotech, Aidenbach, Germany). All other chemicals were of the highest analytical grade and purchased from common sources.

### 2.2. GC-MS Analysis

The chemical composition of the EO was determined employing an Agilent 8890 gas chromatograph (GC) equipped with a single quadrupole 5977B mass spectrometer (Santa Clara, CA, USA) and an autosampler PAL RTC120 (CTC Analytics AG, Zwingen, Switzerland). The separation of the compounds was obtained with an HP-5 capillary column (30 m, 250 μm i.d., 0.25 μm film thickness). The analytical parameters as well as the identification of the components of the EO followed those previously published [29].

### 2.3. Cell Culture

Human neuroblastoma SH-SY5Y (ATCC, Manassas, VA, USA) cell lines were maintained at 37 °C in an incubator in a humidified atmosphere of 5% CO_2_/95% air. The cells were cultured in DMEM high-glucose supplemented with 10% fetal bovine serum (FBS), and 1% (*v*/*v*) antibiotic solution penicillin-streptomycin. The cells were seeded at the density of 5 × 10^3^ in a 96-well plate and were cultured for 24 h prior to treatments. The cells were used earlier to become confluent (80–90%).

### 2.4. Evaluation of Cytotoxicity Using MTT Assay

The cytotoxic effects of Cd^2+^, Hg^2+^, or Pb^2+^ and BEO were investigated using the MTT (3-(4,5-dimethylthiazol2-yl)-2,5-diphenyl tetrazolium bromide) assay. The cells were grown in DMEM. The cells were then seeded in a 96-well plate at a density of 5 × 10^3^ cells per well and allowed to adhere for 24 h in the CO_2_ incubator at 37 °C with 5% CO_2_. The cells were incubated with different concentrations of Cd^2+^, Hg^2+^, Pb^2+^ (0.25, 2.5, 25, or 250 μM), or BEO (0.01–0.03% *v*/*v*) for 24 h. The selected metal concentrations were based on previous studies evaluating their neurotoxic effects on SH-SY5Y cells, ensuring a dose–response range that includes subtoxic, moderate, and highly cytotoxic concentrations [15,17]. Subsequently, 20 μL of MTT stock solution (5 mg/mL in PBS 1X) was added to each well in 180 μL of medium and incubated in the dark. The absorbance was recorded at 540 nm using a multimode multi-well plate reader (Victor 3 Multilabel Microplate Reader (PerkinElmer, Waltham, MA, USA), and cell viability was calculated using the following formula: Growth Inhibition = ((AB540C − AB540T)/AB540C) × 100%. AB540C is the absorbance of untreated cells. AB540T is the absorbance of cells treated with BEO and heavy metal.

### 2.5. Evaluation of Cell Viability by Means of Calcein-AM Assay

Cell viability was measured through the calcein-AM assay (Invitrogen—cod. C3100MP) following the manufacturer’s instructions. Briefly, SH-SY5Y cells were seeded in 96 multi-well plates at a density of 5 × 10^3^ cells/well in the growth medium and allowed to attach overnight. The medium was discarded, and cells were treated with heavy metals diluted in cell growth medium for 24 h at 37° C, 5% CO_2_. The cells incubated in the growth medium without any treatment were used as the control. After 24 h of exposure, the treatments were discarded and SH-SY5Y cells were incubated with 1 μM calcein-AM in PBS for 45 min at 37 °C, and both brightfield and fluorescence images were acquired through a Nikon Digital SIGHT camera and the NIS Elements software (version 3.00 SP7; Nikon, Turin, Italy), with 100× magnification (TE2000 inverted microscope by Nikon, Turin, Italy).

### 2.6. Lactate Dehydrogenase (LDH) Release

Lactate dehydrogenase (LDH) leakage into the culture medium was assessed as a marker of cytotoxicity. To obtain a cell-free supernatant, the culture medium was collected and centrifuged at 3000 rpm for 5 min following exposure to Cd^2+^, Hg^2+^, Pb^2+^, or BEO. The resulting supernatant was then used to determine LDH activity. Briefly, the assay was performed by mixing 0.1 mL of the cell-free supernatant with 48 mM potassium phosphate buffer (pH 7.5), containing 0.18 mM NADH and 0.6 mM sodium pyruvate, in a final volume of 3 mL. A microplate spectrophotometer system (Bio-Rad-680, Bio-Rad, Redmond, WA, USA) was used to measure the change in absorbance at 440 nm. The formula for calculating cell LDH release (% control) is as follows: % control = (U LDH/mg cell protein) treatment/(U LDH/mg cell protein) control 100% [30].

### 2.7. Measurement of Intracellular ROS Production

ROS generation was measured using an oxidation-sensitive fluorescent probe, 2′,7′-dichlorodihydrofluorescein diacetate (DCFH-DA), following a slightly modified protocol from the literature [31]. In brief, SH-SY5Y cells were seeded into a 96-black-well plate and incubated for 24 h. After pre-treatment with the indicated concentrations of BEO (0.01–0.03% *v*/*v* μM) for 24 h, the cells were treated with Cd^2+^, Pb^2+^, or Hg^2+^ (25 μM) for 6 h or H_2_O_2_ (50 μM) for 30 min. Then, they were tested using DCFH-DA at a concentration of 25 μM added in the dark and incubated at 37° for 30 min. The formation of fluorescent dichlorofluorescein (DCF) due to the oxidation of DCFH in the presence of ROS was read at excitation and emission wavelengths of 485 and 530 nm, respectively, using a microplate reader Tecan Infinite M1000 Pro (Tecan, Cernusco S.N., Italy).

### 2.8. Antibacterial Studies

Minimum inhibitory concentrations (MICs, %*v*/*v*) of BEO were determined using the broth microdilution method, following Clinical and Laboratory Standards Institute (CLSI) guidelines (2018) [32,33]. A stock solution of BEO was prepared and serially diluted two-fold in Cation-Adjusted Mueller Hinton Broth (Oxoid, Milan, Italy) to generate a range of concentrations. The following bacterial strains from the ATCC collection were available as freeze-dried discs: Gram-positive strains such as *Staphylococcus aureus* (ATCC 25923, 29213, and 43300) and *Enterococcus faecalis* ATCC 29212, and Gram-negative ones such as *Escherichia coli* ATCC 25922 and *Klebsiella pneuomoniae* (ATCC 13883, 70063). The bacterial strains were maintained as frozen stocks at −80 °C. Pre-cultures were grown in Mueller Hinton Broth (MHB) at 37 °C for 3–5 h. Bacterial suspensions were adjusted to a 0.5 McFarland standard (OD625 nm 0.08–0.10) using a spectrophotometer, as per CLSI procedure M7-A9. The standardized suspension was then further diluted (1:100) with MHB to reach 1–2 × 10^6^ CFU/mL. A 200 μL aliquot of the final inoculum was inserted into each well. As a control for growth, several wells containing only inoculated broth were prepared. The plates were incubated at 37 °C for 24 h, and the MIC values were calculated as the lowest concentration of compounds at which there was no optically detectable bacteria growth. Each experiment was carried out in duplicate and repeated three times to determine the MICs. Levofloxacin (Sigma-Aldrich S.p.a., Milan, Italy) served as the study’s standard reference antibiotic.

### 2.9. Statistical Analysis

All the data are presented as mean ± standard deviation (SD), with values derived from a minimum of three independent experiments. All the assays were performed in triplicate to ensure reproducibility. Statistical significance (*p* < 0.0001) was assessed using a one-way ANOVA followed by Dunnett’s post-hoc test in GraphPad Prism 9.0. Statistical significance was defined as a *p*-value ≤ 0.05.

## 3. Results

### 3.1. GC-MS Analysis

The GC-MS analysis of BEO led to the identification of 99.77% of the total composition (Table 1). In detail, the EO was mainly dominated by monoterpene hydrocarbons (54.31%), followed by oxygenated monoterpenes (45.29%), and by sesquiterpene hydrocarbons (0.17%). Among monoterpene hydrocarbons, limonene resulted in the main compound (38.24%), followed by γ-terpinene (6.58%), and β-pinene (5.76%). On the other hand, linalool acetate (30.50%) and linalool (14.45%) were the main representatives of esters and oxygenated monoterpenes, respectively.

### 3.2. Effects of Cd^2+^, Hg^2+^, Pb^2+^, and BEO on Cell Viability

**Figure 1:**
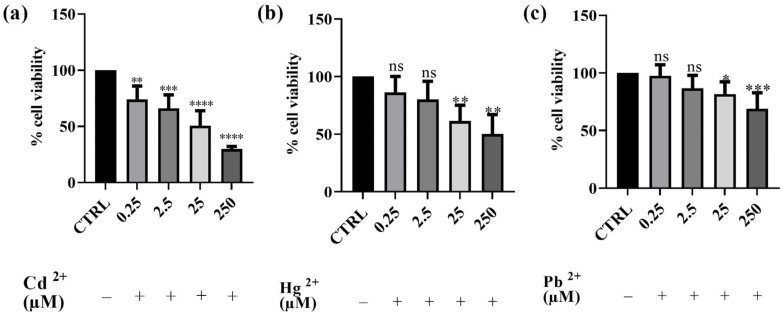
Effects on SH-SY5Y of Cd^2+^ (**a**), Hg^2+^ (**b**), and Pb^2+^ (**c**) (0.25–250 μM) cell viability after 24 h of exposure. Results are shown as mean ± standard deviation (SD) (n = 3). Significant differences versus the control (CTRL): non-significant differences (ns, *p* > 0.05), * *p* < 0.05, ** *p* < 0.01, *** *p* < 0.001, and **** *p* < 0.0001.

Preliminary cytotoxicity assays were conducted to assess the impact of Cd^2+^, Hg^2+^, and Pb^2+^ on human SH-SY5Y neuronal cells. The results obtained are depicted in Figure 1. The effect of each of the three metals on cell viability was evaluated using the MTT assay. After 24 h of exposure to different concentrations of Cd^2+^, Hg^2+^, and Pb^2+^, a dose-dependent cytotoxic effect was observed in the neuronal model. Among the three metals tested, Cd^2+^ exhibited the highest toxicity, followed by Hg^2+^, whereas Pb^2+^ was the least toxic. Cd^2+^ caused the most significant reduction in cell viability at the highest concentration, followed by Hg^2+^, while Pb^2+^ showed the least toxicity. Indeed, after treatment with Cd^2+^, the cell survival rate decreased from 74%, at the lowest concentration of 0.25 μM, to 31% at the concentration of 250 μM. Hg^2+^ toxicity was observed to be intermediate, with cell viability decreasing from 86 to 50% at the lowest and the highest concentrations tested, respectively. In contrast, Pb^2+^ did not significantly affect cell viability at lower concentrations (0.25 and 2.5 μM); however, at the highest concentration tested (250 μM), cell viability decreased to 68%. The findings obtained with this first set of experiments enabled us to select the concentration of metals that notably reduced viability for testing the potential protective effect of BEO. Consequently, the concentration of 25 μM was selected for subsequent experiments as it induced significant cytotoxicity without completely affecting the cell population. This concentration was chosen to ensure that the metals’ toxic effects were measurable while still maintaining a sufficient number of viable cells for evaluating potential protective effects. Specifically, at 25 μM, Cd^2+^ led to the most substantial reduction in cell viability (approximately 50%), making it the most toxic metal in our model, whereas Pb^2+^, the least toxic metal, induced the first detectable significant decrease in viability, thus allowing for a reliable assessment of both toxicity and potential cytoprotection in later experiments.

**Figure 2:**
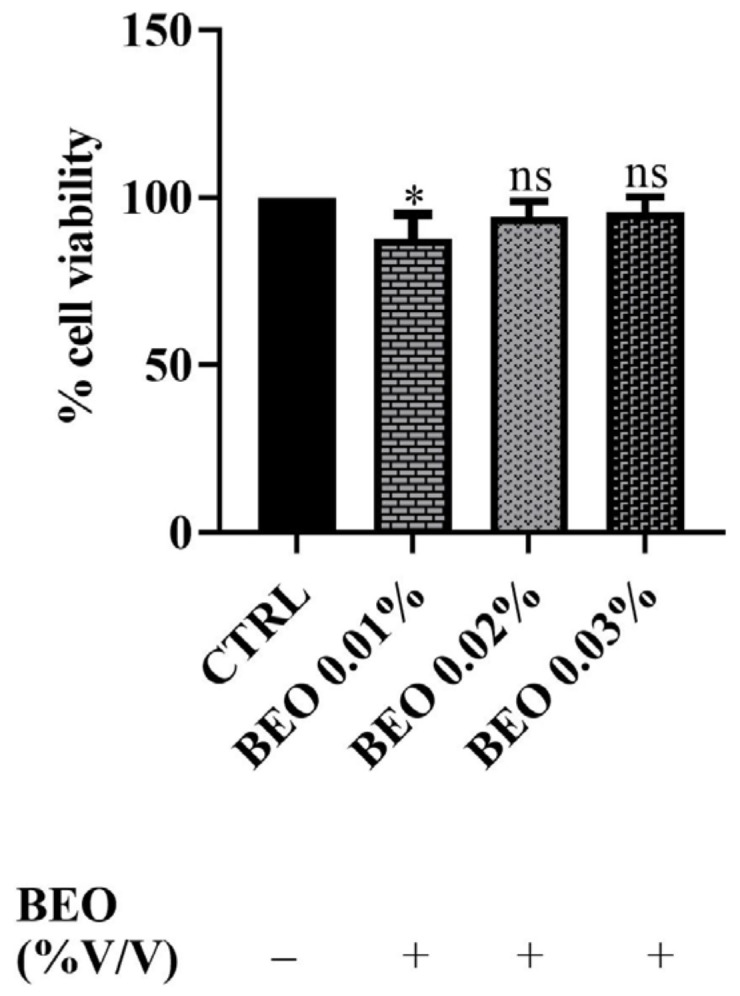
Effects of BEO on SH-SY5Y (0.01, 0.02, and 0.03% *v*/*v*) cell viability after 24 h of exposure. Results are shown as mean ± standard deviation (SD) (n = 3). Significant differences versus the control (CTRL): non-significant differences (ns, *p* > 0.05) and * *p* < 0.05.

In addition, SH-SY5Y cells were exposed to concentrations of 0.01, 0.02, and 0.03 (% *v*/*v*) of BEO for a 24 h incubation period. The results, reported in Figure 2, demonstrated that BEO treatment did not significantly affect cell viability. Indeed, the data showed no statistically significant reduction in the metabolic activity in BEO-treated cells compared to untreated controls at the concentrations of 0.02 and 0.03%. Surprisingly, at the lowest concentration (0.01%), a slight decrease in cell viability was observed (amounting to 88%). This phenomenon may be explained by the hormetic effect, where low concentrations of bioactive compounds induce a mild cellular stress response, while higher concentrations activate protective mechanisms. Additionally, certain components of BEO, such as linalool and limonene, have been reported to induce cell cycle arrest in some cellular models, that could account for the observed reduction in cell viability [37].

### 3.3. Effect of BEO on Cd^2+^-, Hg^2+^-, and Pb^2+^-Induced Cytotoxicity

**Figure 3:**
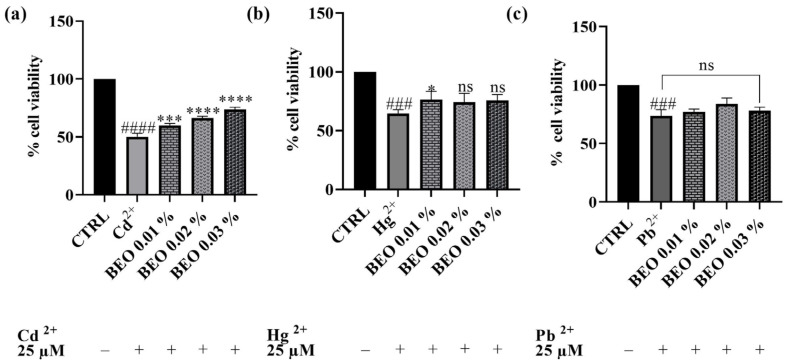
Effects on SH-SY5Y cell viability of Cd^2+^ (**a**), Hg^2+^ (**b**), and Pb^2+^ (**c**), at the concentration of 25 μM, and BEO (0.01, 0.02, and 0.03% *v*/*v*) after 24 h of co-treatment. Results are shown as mean ± standard deviation (SD) (n = 3). Significant differences versus Cd^2+^, Hg^2+^, or Pb^2+^: non-significant differences (ns, *p* > 0.05), * *p* < 0.05, *** *p* < 0.001, and **** *p* < 0.0001. Significant differences versus control (CTRL): ### *p* < 0.001 and #### *p* < 0.0001.

After establishing the non-toxic nature of the tested BEO concentrations on the SH-SY5Y cell line, subsequent experiments were conducted to assess their potential cytoprotective properties against cell damage induced by exposure to Cd^2+^, Hg^2+^, and Pb^2+^ (25 μM). As shown in Figure 3, treatment with 25 μM of heavy metals significantly reduced cell viability compared to the control group, indicating substantial cytotoxicity. Co-treatment with BEO effectively attenuated Cd^2+^-induced cytotoxicity in a concentration-dependent manner, with cell viability increasing to 74% at the highest BEO concentration (0.03%) compared to 50% in Cd^2+^-only treated cells. In the case of Hg^2+^, a significant protective effect was observed only at the lowest BEO concentration (0.01%), while higher concentrations did not yield statistically significant improvements in cell viability. Conversely, no significant protective effect of BEO was detected in Pb^2+^-treated cells, which aligns with the lower intrinsic toxicity of this metal under the tested conditions.

**Figure 4:**
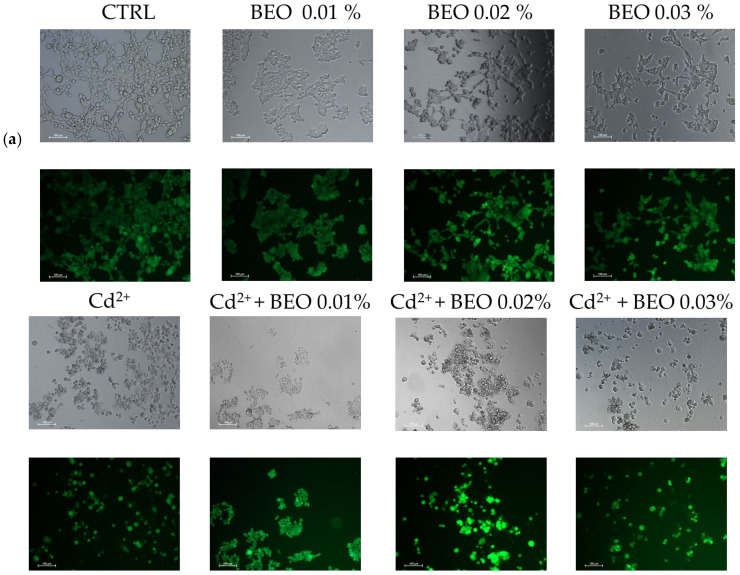

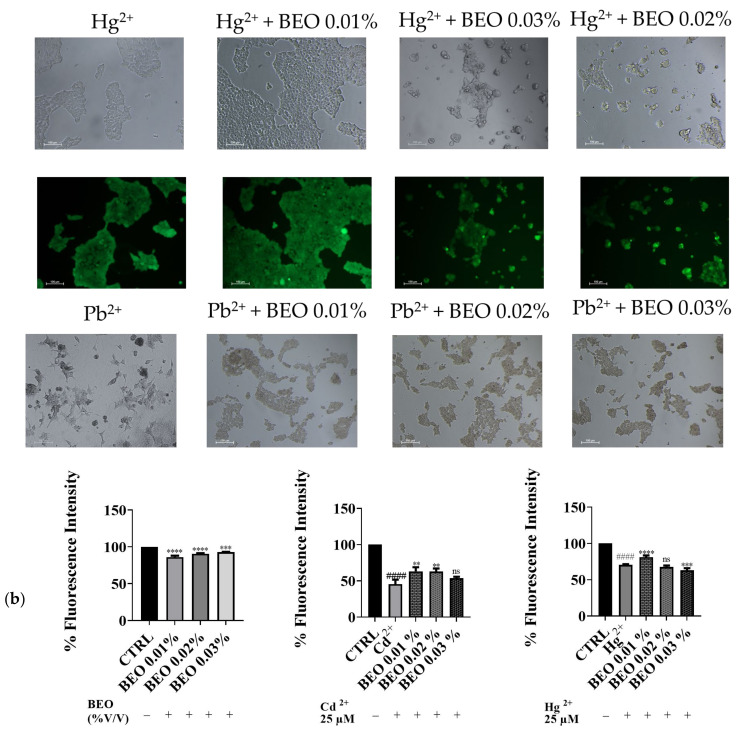
Effect of BEO on cytotoxicity induced by heavy metals in SH-SY5Y cells. (**a**) Representative brightfield and fluorescence microscopy images showing SH-SY5Y cells treated with 25 μM Cd^2+^, Hg^2+^, or Pb^2+^, 0.01, 0.02, or 0.03% BEO, or co-treated with the three metals and the three BEO concentrations. Brightfield images depict cellular morphology, while fluorescence images (calcein AM staining) indicate viable cells. Images were acquired using a Nikon Digital SIGHT camera at 100× magnification. (**b**) Quantification of fluorescence intensity relative to the control group. Results are shown as mean ± standard deviation (SD) (n = 3). Significant differences versus Cd^2+^, Hg^2+^, or Pb^2+^: non-significant differences (ns, *p* > 0.05), ** *p* < 0.01, *** *p* < 0.001, and **** *p* < 0.0001. Significant differences versus control (CTRL): #### *p* < 0.0001.

To confirm the cytoprotective assay of BEO against metals toxicity, the calcein-AM assay, a widely used method for assessing cell viability, was carried out. In this assay, live cells with intact esterases convert the non-fluorescent calcein-AM into the highly fluorescent calcein, resulting in green fluorescence. Our results demonstrated that exposure to Cd^2+^, Hg^2+^, and Pb^2+^ (25 μM) significantly decreased cell viability, as indicated by reduced green fluorescence (Figure 4a). However, no detectable fluorescence was observed in cells treated with Pb^2+^, suggesting that Pb^2+^ may interfere with the calcein-AM assay or induce cell death through a mechanism without involving esterase activity. Simultaneous treatment with BEO at concentrations of 0.01, 0.02, and 0.03% attenuated the cytotoxic effects of Cd^2+^, as evidenced by the increased green fluorescence in the treated groups compared to the Cd^2+^-only group. In contrast, for Hg^2+^, a protective effect was observed only at the lowest BEO concentration (0.01%), while higher concentrations did not lead to a significant increase in fluorescence. These findings suggest that BEO exerts cytoprotective properties against Cd^2+^-induced toxicity in a concentration-dependent manner, while its effect on Hg^2+^ toxicity appears to be more limited. The optimal protective concentration of BEO varied depending on the specific metal, highlighting the need for a tailored approach when considering BEO as a potential therapeutic agent against heavy metal toxicity.

### 3.4. Effects of BEO on Cd^2+^, Hg^2+^, and Pb^2+^-Induced LDH Release

**Figure 5:**
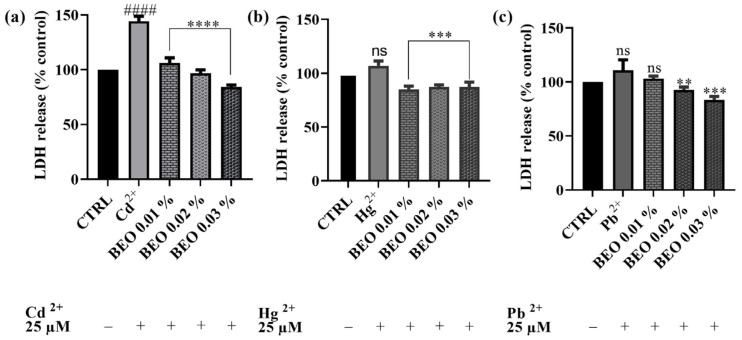
LDH activity of SH-SY5Y cell after exposure for 24 h to Cd^2+^ (**a**), Hg^2+^ (**b**), or Pb^2+^ (**c**) and concentrations of BEO (0.01–0.03% *v*/*v*). Data are expressed as mean ± SD (n = 3). Significant differences versus Cd^2+^, Hg^2+^, or Pb^2+^: non-significant differences (ns, *p* > 0.05), ** *p* < 0.01, *** *p* < 0.001, and **** *p* < 0.0001. Significant differences versus control (CTRL): non-significant differences (ns, *p* > 0.05), #### *p* < 0.0001.

To investigate the protective effects of BEO against heavy metal-induced membrane damage, the LDH assay was performed on SH-SY5Y cells. This assay measures the release of LDH, a stable cytosolic enzyme, into the culture medium, which occurs as a result of cell membrane damage caused by apoptosis, necrosis, or other cytotoxic events. As shown in Figure 5, BEO significantly attenuated Cd^2+^-induced LDH release, confirming its protective effects. Notably, BEO exhibited the most pronounced protective effect against Cd^2+^ cytotoxicity, with a significant reduction in LDH release in a concentration-dependent manner. At the highest BEO concentration (0.03%), Cd^2+^-induced LDH release was reduced by approximately 40%. In contrast, Hg^2+^ exposure did not significantly enhance LDH release compared to the control, suggesting that Hg^2+^ toxicity may not involve substantial membrane damage detectable by this assay. However, treatment with BEO at 0.02% and 0.03% resulted in a modest reduction in LDH release by approximately 10% compared to the Hg^2+^-exposed cells, indicating a potential protective effect. Similarly, Pb^2+^ exposure did not lead to a significant increase in LDH release, yet BEO treatment decreased LDH levels by approximately 8%, 18%, and 28% at concentrations of 0.01%, 0.02%, and 0.03%, respectively. These findings indicate that BEO effectively mitigates Cd^2+^-induced cytotoxicity, while its effects on Hg^2+^ and Pb^2+^ require further investigation to determine their biological significance.

### 3.5. Scavenging Effects of BEO Against Cd^2^-, Hg^2+^-, Pb^2+^-, and H_2_O_2_ -Induced Oxidative Stress

**Figure 6:**
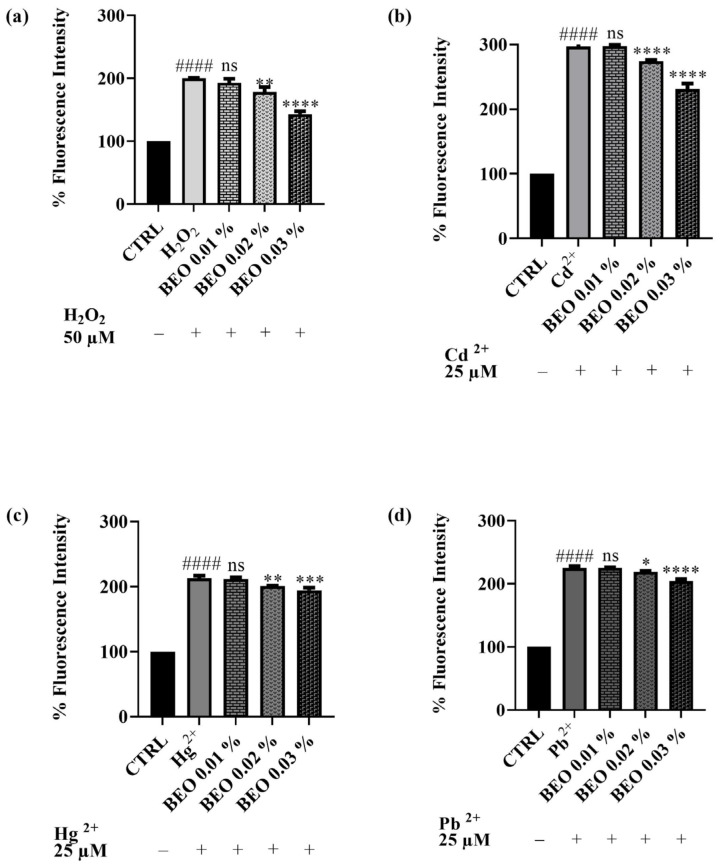
DCFH oxidation in SH-SY5Y cells after exposure to H_2_O_2_, 50 μM (**a**), Cd^2+^ (**b**), Pb^2+^ (**c**), or Hg^2+^ (**d**) (25 μM) and different concentrations of BEO (0.01–0.03% *v*/*v*). Results are shown as mean ± standard deviation (SD) (n = 3). Significant differences versus H_2_O_2_, Cd^2+^, Hg^2+^, or Pb^2+^: non-significant differences (ns, *p* > 0.05), * *p* < 0.05, ** *p* < 0.01, *** *p* < 0.001, and **** *p* < 0.0001. Significant differences versus control (CTRL): #### *p* < 0.0001.

The potential of BEO to attenuate oxidative stress triggered by heavy metals or hydrogen peroxide in SH-SY5Y cells has been evaluated. The antioxidant activity of BEO was quantified in vitro using the 2′,7′-dichlorodihydrofluorescein diacetate (DCFH-DA) assay in a cellular model. This assay measures the compound efficacy to inhibit the oxidation of 2′,7′-dichlorodihydrofluorescein (DCFH) to its fluorescent oxidized product, 2′,7′-dichlorofluorescein (DCF). The capacity of BEO to prevent ROS production induced by H_2_O_2_ (Figure 6a) in the neuronal cells has been assessed. H_2_O_2_ was used at a concentration of 50 μM. The results demonstrated that BEO (0.01–0.03% *v*/*v*) reduced oxidative stress induced by H_2_O_2_ in a dose-dependent manner. The co-treatment of cells with H_2_O_2_ and BEO 0.03% produced a decrease of fluorescence intensity of 35% with respect to H_2_O_2_-only-treated cells. A similar trend was observed for cells exposed to the heavy metals (used at a concentration of 25 μM). Specifically, noteworthy results were achieved when cells were co-treated with BEO and Cd^2+^, which has been identified as the metal that caused the highest production of cellular ROS in this assay. Indeed, ROS levels induced by Cd^2+^ were significantly decreased by BEO at the highest concentration. A similar effect was observed when ROS production was induced by Hg^2+^ or Pb^2+^. Even in these cases, the protective effect of BEO towards ROS production was found to be dose-dependent.

### 3.6. Antibacterial Effects of BEO

BEO was evaluated for its antibacterial activity in accordance with the Clinical Laboratory Standards Institute (CLSI) recommendations [33]. The broth microdilution method was applied to evaluate its inhibitory growth effects against Gram-positive (*S. aureus* 25923, *S. aureus* 29213, *S. aureus* 43300, and *E. faecalis* 29212) and Gram-negative (*E. coli* 25922, *K. pneumoniae* 13883, and *K. pneumoniae* 70063) bacteria belonging to the ATCC collection using levofloxacin as a reference drug. Table 2 lists the results expressed as MIC (% *v*/*v* for BEO and μg/mL for levofloxacin). These data are particularly interesting because they demonstrate the BEO broad antibacterial activity across all the tested bacteria, with slight variations in efficacy depending on the strain. For Gram-positive bacteria, the MIC values ranged from 0.5 to 2%. Notably, *S. aureus* ATCC 29213 and *S. aureus* ATCC 25923 exhibited the lowest MIC values of 0.5%, indicating a high susceptibility to BEO. In contrast, *S. aureus* ATCC 43300, a methicillin-resistant strain (MRSA), showed a slightly higher MIC of 1%, while *E. faecalis* ATCC 29212 exhibited the highest MIC among Gram-positive bacteria at 2%. For Gram-negative bacteria, MIC values were generally higher, with *E. coli* ATCC 25922 showing an MIC of 1%, while *E. coli* ATCC 35218 required 2% for inhibition. *K. pneumoniae* strains demonstrated the highest MIC values, with *K. pneumoniae* ATCC 13883 and *K. pneumoniae* ATCC 70063 both exhibiting MICs of 4%. These results confirm that BEO exhibits stronger antibacterial activity against Gram-positive bacteria, particularly *S. aureus*, while Gram-negative bacteria, especially *K. pneumoniae*, require higher concentrations for effective inhibition. This differential activity could be attributed to the structural differences between the cell walls of Gram-positive and Gram-negative bacteria, with the latter outer membrane potentially limiting the penetration of BEO.

## 4. Discussion

Exposure to heavy metals poses a significant risk to human health, particularly due to their long-lasting neurotoxic effects. Cd^2+^, Hg^2+^, and Pb^2+^ contribute to oxidative stress, mitochondrial dysfunction, and neuroinflammation, all of which are critical factors in neurodegenerative disorders, among which is AD [10,11,12,13]. These metals disrupt antioxidant defense systems, impair cellular energy production, and alter immune responses, exacerbating neuronal damage [15,18].

In addition to direct neuronal damage, heavy metals profoundly affect the glial compartment, particularly microglia and astrocytes, which play a crucial role in maintaining brain homeostasis. Microglia, the resident immune cells of the central nervous system (CNS), respond to toxic insults by adopting a pro-inflammatory phenotype, releasing cytokines, such as interleukin-1β (IL-1β), tumor necrosis factor-alpha (TNF-α), and interleukin-6 (IL-6) [38]. This persistent neuroinflammatory state exacerbates neuronal dysfunction and death, contributing to the progression of neurodegenerative diseases. Similarly, astrocytes, which regulate synaptic function and antioxidant defense, undergo reactive astrogliosis in response to heavy metal exposure, leading to further oxidative stress and the disruption of neuronal support mechanisms [39]. Modulating glial cell activity and reducing neuroinflammation are therefore considered promising strategies for neuroprotection. In this context, natural compounds, including essential oils as BEO, with anti-inflammatory and antioxidant properties may help restore cellular homeostasis, potentially mitigating the detrimental effects of toxic insults on the central nervous system [20]. Given the role of glial cells in neuroinflammation and oxidative stress, it would be particularly interesting to explore how BEO might influence their activation and function. Indeed, its known anti-inflammatory and antioxidant properties suggest that BEO could modulate microglial activation and astrocyte reactivity, potentially counteracting the detrimental effects of heavy metal toxicity [39,40], suggesting glial cells as a potential target in neuroprotection. In recent years, there has been a growing interest in the search for natural agents capable of counteracting the toxic effects of heavy metals and oxidative stress. Both of these effects could be crucial factors in the onset of neurodegenerative diseases, such as AD. Naturally derived compounds may represent complementary agents to conventional treatments for neurodegenerative disorders [13]. In this regard, BEO has gained attention due to its versatility and recognized multiple properties. However, its neuroprotective potential, particularly in the context of heavy metal toxicity, has remained yet largely unexplored. Despite the promising in vitro findings, the clinical translation of BEO requires a deeper understanding of its bioavailability, metabolism, and potential toxicity. Essential oils are complex mixtures of bioactive compounds that undergo metabolic transformations after administration. In vivo BEO constituents such as limonene and linalool are metabolized primarily in the liver through cytochrome P450 enzymes, leading to the formation of oxygenated metabolites that may have altered biological activity [41]. These metabolites can influence the systemic distribution, half-life, and potential bioefficacy of BEO.

Another critical aspect to consider is the potential systemic toxicity of BEO upon prolonged use. While essential oils are generally regarded as safe at low doses, some studies suggest that chronic exposure to high concentrations may lead to hepatotoxicity or neurotoxic effects [42]. Therefore, further pharmacokinetic and toxicological studies are essential to define the safe therapeutic window for BEO application in neuroprotection.

The present work is an attempt to demonstrate the cytoprotective ability of BEO on SH-SY5Y neuroblastoma cells against heavy metal (Cd^2+^, Hg^2+^, and Pb^2+^) toxicity alongside the evaluation of its antibacterial effects against pathogens of clinical interest.

### 4.1. BEO Chemical Composition

The chemical composition of BEO has been well documented and that reported in this work is consistent with those of other essential oils from the same plant species. Indeed, the main constituents are limonene (25–53%), linalool (2–20%), linalyl acetate (15–40%), γ-terpinene, and β-pinene. Similar chemical compositions have also been found in other works [43]. For instance, Furneri et al. [44] reported limonene (25.65–33.69%), linalyl acetate (20.14–27.06%), β-pinene (4.54–6.00%), and γ-terpinene (7.35–9.65%) as the predominant constituents. Similarly, Rasheed et al. (2024) [45] identified limonene (23.21%) as the dominant component of BEO, followed by linalyl acetate (14.01%) and linalool (9.96%). This chemical composition is maintained also with different distillation times, as demonstrated by Bozova et al. Indeed, the BEO obtained in the above-mentioned study was mainly characterized by limonene, linalool, and linalyl acetate in the range of 42.36–53.25%, 5.59–12.18%, and 20.84–26.73%, respectively [46].

### 4.2. Effects of Cd^2+^, Hg^2+^, Pb^2+^, and BEO on Cell Viability and the Cytoprotective Role of BEO Against Heavy Metals-Induced Cytotoxicity

The preliminary cytotoxicity evaluation conducted by means of the MTT assay in this study provides valuable insights into the neurotoxic effects of Cd^2+^, Hg^2+^, and Pb^2+^ on SH-SY5Y human neuronal cells, as well as the lack of BEO cytotoxicity. As anticipated, the results demonstrated a dose-dependent cytotoxic effect of these heavy metals, with Cd^2+^ being the most potent, followed by Hg^2+^ and Pb^2+^, which showed the lowest toxicity at the tested concentrations. These findings are consistent with existing literature, which has documented the neurotoxic potential of these metals, albeit with varying degrees of severity, through mechanisms involving oxidative stress, mitochondrial dysfunction, and apoptosis [12,47,48]. Indeed, in our study, Cd^2+^ exposure resulted in a significant decrease in cell viability, from 74% at the lowest concentration (0.25 μM) to 31% at 250 μM, corroborating findings from similar studies that report a dose-dependent decline in neuronal cell viability following Cd^2+^ exposure [49]. Hg^2+^ also demonstrated cytotoxic effects in SH-SY5Y cells, with a decrease in cell viability from 86 to 50% at the lowest and highest concentrations, respectively. Hg^2+^ toxicity and its mechanism has been well documented in neuronal cell lines [50]. Pb^2+^, on the other hand, showed the least cytotoxicity in our experiments. Pb^2+^ toxicity has been well documented in the literature, particularly its ability to interfere with calcium homeostasis, disrupt neurotransmitter release, and induce oxidative damage in neural tissue [51]. Notably, BEO itself did not reduce cell viability up to a concentration of 0.03%, in contrast to the findings of Berliocchi et al. [52], who reported toxic effects at this dose in SH-SY5Y cells. Subsequently, the potential of BEO to provide cytoprotection against heavy metal-induced cytotoxicity, at the concentration of 25 μM, in SH-SY5Y neuronal cells has been investigated. Specifically, BEO demonstrated significant protective effects against the toxicity induced by Cd^2+^, where cell viability increased by 74% at the highest tested concentration (0.03%). Notably, in the case of Hg^2+^-induced cytotoxicity, the greatest protective effect was observed at 0.01% BEO, rather than at higher concentrations, suggesting a non-linear concentration–response relationship. The non-linear concentration–response effects have been frequently observed with plant-derived bioactive compounds and can be attributed to multiple factors [53]. One possible explanation is the differential activation of cellular protective mechanisms at distinct BEO concentrations. At lower concentrations, BEO components—particularly limonene and linalool—may effectively enhance endogenous antioxidant defenses, such as the Nrf2/ARE pathway, leading to the increased expression of detoxifying enzymes (e.g., glutathione peroxidase and superoxide dismutase). However, at higher concentrations, these same compounds might saturate cellular detoxification pathways or induce adaptive stress responses that inadvertently compromise cellular homeostasis [54]. Another plausible mechanism involves the interaction between BEO constituents and Hg^2+^ ions. At optimal BEO concentrations, the essential oil bioactive components may efficiently chelate metal ions and mitigate their cytotoxic effects by reducing intracellular accumulation. However, at higher BEO concentrations, increased membrane permeability or altered cellular signaling could inadvertently enhance metal uptake or disrupt ion transport systems, reducing the protective capacity of the oil [55]. Additionally, it is possible that higher BEO concentrations trigger pro-oxidant effects, a phenomenon known as the “hormetic response”, where plant-derived antioxidants act as mild stressors at low doses but contribute to oxidative stress at higher doses. This biphasic effect has been reported for several polyphenolic and terpenoid compounds [33]. Moreover, high concentrations of BEO might interfere with mitochondrial function, altering ATP production and redox homeostasis. While lower doses could enhance mitochondrial efficiency and biogenesis, excessive amounts may lead to mitochondrial uncoupling, increased ROS production, and apoptotic signaling [56]. Finally, it is also possible that high BEO concentrations modulate key signaling pathways involved in cellular stress responses, such as MAPK and NF-κB, in a dose-dependent manner. While low doses may exert anti-inflammatory and cytoprotective effects, higher concentrations might overstimulate these pathways, leading to cellular dysfunction and reduced resilience to Hg^2+^-induced toxicity [57]. MAPK signaling is particularly relevant in metal-induced cytotoxicity, as Cd^2+^ and Hg^2+^ are known to activate ERK1/2 and JNK pathways, leading to oxidative damage and apoptosis. BEO components might counteract this activation by modulating MAPK phosphatases (MKPs) and suppressing excessive JNK phosphorylation. In addition, limonene and linalool have been reported to inhibit NF-κB activation, reducing the expression of pro-inflammatory cytokines, such as TNF-α and IL-6, which are often upregulated in response to heavy metal exposure [58]. Another important pathway potentially involved in BEO’s protective effects is the PI3K/Akt signaling pathway, which plays a crucial role in cell survival and neuroprotection. Preliminary evidence suggests that linalool and limonene can enhance Akt phosphorylation, thereby promoting cell survival and resistance to oxidative stress-induced apoptosis [59]. This mechanism could explain the observed increase in cell viability at specific BEO concentrations.

The substantial protective effect of BEO observed in this study aligns with evidence suggesting that the essential oil principal bioactive components (see Table 1), such as limonene and linalool, can chelate metal ions and neutralize ROS, thereby reducing oxidative stress [60,61]. The calcein-AM assay further supported the cytoprotective effects of BEO, particularly against Cd^2+^ and Hg^2+^ toxicity. The reduced green fluorescence observed in cells exposed to Pb^2+^ suggests that Pb^2+^ itself may interfere with esterase activity or induce cell death via alternative mechanisms that are not detectable by this assay. This finding is consistent with studies suggesting that Pb^2+^ toxicity often involves interference with Cd^2+^ signaling and ion channel function, mechanisms that may not directly impact esterase activity [62]. Moreover, Pb^2+^ is known to bind sulfhydryl (-SH) groups of enzymatic proteins, leading to enzyme inhibition. Since calcein-AM fluorescence depends on active intracellular esterases converting the non-fluorescent calcein-AM into fluorescent calcein, Pb^2+^-induced inhibition of these enzymes could explain the reduced fluorescence signal. Indeed, previous studies have shown that heavy metals, including Pb^2+^, can suppress esterase and protease activities, thereby altering cellular function and viability [63].

### 4.3. Effects of BEO on Cd^2+^, Hg^2+^, and Pb^2+^-Induced LDH Release and Oxidative Stress

The LDH assay results further corroborate the protective effects of BEO against heavy metal-induced cytotoxicity in SH-SY5Y neuronal cells. LDH release is a well-established marker of cell membrane damage caused by cytotoxic events, including apoptosis and necrosis. In this study, co-treatment with BEO significantly reduced LDH release in cells exposed to Cd^2+^, Hg^2+^, and Pb^2+^, demonstrating its ability to mitigate membrane damage and preserve cell integrity. Notably, BEO exhibited once again the most pronounced protective effect against Cd^2+^-induced cytotoxicity, as evidenced by a 40% reduction in LDH release at the highest concentration tested. The reduction in LDH release observed in this study supports the hypothesis that BEO, rich in antioxidant compounds such as limonene and linalool, scavenges ROS and reduces oxidative stress, thereby preventing Cd^2+^-induced membrane damage. The clear concentration-dependent effect suggests that at higher concentrations, BEO may more effectively counteract lipid peroxidation and stabilize the cell membrane, possibly through the enhanced activation of antioxidant defense mechanisms. The protective effect of BEO against Hg^2+^-induced membrane damage, while significant, was less pronounced and did not exhibit a clear concentration-dependent trend. This observation may reflect differences in the mechanism of toxicity between Cd^2+^ and Hg^2+^. The latter is known to exert its cytotoxic effects by binding to thiol groups in proteins and disrupting cellular enzymatic functions, potentially causing membrane damage indirectly. This irreversible binding to critical sulfhydryl-containing proteins may limit the protective potential of BEO, as even effective antioxidant defenses might not be sufficient to counteract Hg^2+^-induced damage at higher concentrations [64].The lack of a concentration-dependent protective effect of BEO against Hg^2+^ suggests that while its antioxidant properties may counteract some aspect of the oxidative damage, other mechanisms of Hg^2+^-induced cytotoxicity may not be fully mitigated by BEO. Indeed, another possible explanation is that higher concentrations of BEO could lead to slight alterations in membrane fluidity, which might impact its protective capacity against Hg^2+^ toxicity in a non-linear manner. In the case of Pb^2+^-induced cytotoxicity, BEO demonstrated a moderate but consistent protective effect, with LDH release showing a clear concentration-dependent reduction. This result is noteworthy as Pb^2+^ toxicity is often associated with a disruption in calcium signaling and oxidative stress, which contribute to membrane destabilization [65]. Moreover, Pb^2+^ is known to mimic calcium ions, disrupting synaptic signaling and neurotransmitter release, mechanisms that may not be effectively counteracted by antioxidant activity alone. Additionally, Pb^2+^ has a high affinity for sulfhydryl (-SH) groups in proteins, leading to irreversible inhibition of critical enzymes such as δ-aminolevulinic acid dehydratase (ALAD), which is essential for heme biosynthesis. This enzyme inhibition could contribute to altered cellular energy metabolism and increased vulnerability to Pb^2+^ toxicity, which may not be significantly mitigated by BEO treatment [66]. When compared with findings in the literature, the results of this study are consistent with previous research demonstrating the protective potential of plant-derived antioxidants against heavy metal-induced toxicity [67]. The ability of BEO to reduce LDH release in a concentration-dependent manner suggests that its protective effect may involve multiple mechanisms among which stabilization of the cell membrane and attenuation of oxidative stress. Indeed, oxidative stress is a critical mechanism contributing to neurodegeneration induced by Cd^2+^, Hg^2+^, and Pb^2+^. The results of this study provide compelling evidence that BEO exerts significant antioxidant effects in SH-SY5Y neuronal cells, reducing ROS production induced by both hydrogen peroxide (H_2_O_2_) and heavy metals. BEO demonstrated a dose-dependent reduction in ROS levels when cells were exposed to H_2_O_2_, suggesting its strong potential to counteract general oxidative stress mechanisms. These findings align with previous studies reporting the antioxidant activity of BEO, primarily attributed to its bioactive components, such as limonene and linalool, which have been shown to scavenge free radicals and enhance cellular antioxidant defenses [68].

When applied to cells exposed to heavy metals, BEO effectively attenuated ROS production induced by Cd^2+^, Hg^2+^, and Pb^2+^, with the most pronounced effect observed in Cd^2+^-treated cells. Cd^2+^ is known to generate ROS through the Fenton-like reaction and disruption of mitochondrial function, leading to oxidative stress that overwhelms cellular defenses [69]. The ability of BEO to reduce significantly decrease Cd^2+^-induced ROS levels is consistent with previous observation reporting similar reductions in Cd^2+^-induced oxidative stress using plant-derived antioxidants [70]. For Hg^2+^ and Pb^2+^, which induced lower ROS production compared to Cd^2+^, BEO also demonstrated dose-dependent antioxidant effects. Although the protective effect of BEO against Hg^2+^ and Pb^2+^ toxicity was less pronounced, these results are still significant given the chronic oxidative stress associated with these metals. Hg^2+^ is known for its ability to bind to thiol groups in proteins and disrupt redox balance, while Pb^2+^ primarily interferes with calcium signaling and induces lipid peroxidation [67]. The findings support the hypothesis that BEO acts by neutralizing ROS and preventing oxidative damage, contributing to its cytoprotective effects observed in previous assays, such as the LDH assay. Moreover, the greater efficacy of BEO against Cd^2+^-induced oxidative stress compared to Hg^2+^ and Pb^2+^ may reflect differences in the underlying toxicity mechanisms of these metals and their interaction with BEO bioactive components. In conclusion, this study highlights the potent antioxidant properties of BEO in mitigating heavy metal-induced oxidative stress in neuronal cells. These results are consistent with existing literature on the antioxidant effects of plant-derived essential oils and underscore BEO potential as a neuroprotective agent.

### 4.4. Antibacterial Effects of BEO

The results of this study demonstrate that BEO exhibits notable antibacterial activity, particularly against Gram-positive bacteria, including methicillin-resistant *S. aureus* (MRSA). These findings are consistent with the well-documented antimicrobial properties of essential oils, which are primarily attributed to their bioactive components, such as monoterpenes (e.g., limonene) and oxygenated derivatives (e.g., linalool), that disrupt bacterial membranes and interfere with essential cellular processes [71]. The MIC values obtained for Gram-positive bacteria ranged from 0.5 to 2% (*v*/*v*), with *S. aureus* ATCC 29213 and *E. faecalis* 29212 being the most susceptible strains. The observed activity of BEO against MRSA (*S. aureus* 43300) is particularly significant, as MRSA infections pose a major challenge due to their resistance to conventional antibiotics. Previous studies have similarly reported the efficacy of essential oils against MRSA, suggesting their potential as adjunctive treatments to combat antibiotic resistance [72]. Although Gram-negative bacteria, such as *E. coli* and *K. pneumoniae*, exhibited higher MIC values (2–4%), indicating reduced susceptibility to BEO, these results remain significant. Indeed, Gram-negative pathogens are of particular concern in healthcare settings due to their pathogenicity and increasing multidrug resistance. Despite the higher MIC values, BEO still demonstrated inhibitory effects, highlighting its potential against these difficult-to-treat microorganisms. This reduced efficacy in Gram-negative bacteria can likely be attributed to their outer membrane structure, which acts as a barrier to the penetration of hydrophobic compounds like essential oils [73]. However, despite requiring higher concentrations, BEO still demonstrated inhibitory effects against these pathogens, which are often associated with healthcare-associated infections and exhibit multidrug resistance. In particular, the importance of these results lies in the fact that it is rare to find natural substances that are effective against strains like *K. pneumoniae*, which is often resistant to conventional antibiotic treatments [74]. This intriguing antibacterial activity could be due to the rich chemical composition of BEO. In fact, the literature suggests that the constituents of essential oils act synergistically, enhancing their overall antibacterial effects [75]. The findings of this study also underscore the therapeutic potential of BEO as a natural antibacterial agent, particularly against Gram-positive pathogens, including drug-resistant strains like MRSA. However, the relatively higher MIC values for Gram-negative bacteria highlight the need for further optimization, such as exploring synergistic effects with conventional antibiotics or repositioned drugs. Previous studies have shown that essential oils can enhance the efficacy of antibiotics or non-antibiotic drugs by disrupting bacterial membranes and facilitating drug uptake [76]. Thus, combining BEO with antibiotics could represent a promising strategy for overcoming bacterial resistance.

## 5. Conclusions

The findings of this work highlight the promising neuroprotective and antibacterial properties of BEO in the context of heavy metal-induced toxicity and bacterial infections. While essential oils have been extensively studied for their antioxidant and antimicrobial properties, to our knowledge, the protective effects of BEO against heavy metal-induced neurotoxicity have never been explored. This work provides novel insights into the cytoprotective effects of BEO, demonstrating its ability to attenuate oxidative stress, reduce cellular damage, and improve neuronal cell viability in the presence of Cd^2+^, Hg^2+^, and Pb^2+^. This cytoprotective activity was especially notable against Cd^2+^, a highly toxic heavy metal with well-documented neurotoxic properties. Furthermore, our study demonstrated the effectiveness of BEO in mitigating oxidative stress induced by both H_2_O_2_ and heavy metals, with a particularly pronounced effect against Cd^2+^-induced oxidative stress. In addition, the antibacterial activity of BEO is noteworthy, particularly against Gram-positive bacteria, such as methicillin-resistant *S. aureus* (MRSA), and Gram-negative strains, like *K. pneumoniae*. The significance of these findings lies in the fact that *K. pneumoniae* is a highly resistant bacterial strain. Thus, identifying natural substances capable of inhibiting such pathogens remains a substantial challenge in antimicrobial research. The strength of this study lies also in its multidisciplinary approach, which integrates cytotoxicity, oxidative stress, and antibacterial assays to comprehensively evaluate the BEO protective and therapeutic effects. The use of SH-SY5Y neuronal cells provides a relevant model for studying neurotoxic mechanisms, while the incorporation of multiple experimental techniques (MTT, calcein-AM, LDH, and DCFH-DA assays) strengthens the validity of these findings. However, the lack of in vivo data limits the translation of these results to clinical or real-world applications. Further in vivo studies are essential to confirm the efficacy, bioavailability, and safety of BEO under physiological conditions. Moreover, it could be interesting to evaluate the potential synergistic effects of BEO with other therapeutic agents, such as antibiotics or antioxidants, which could enhance its efficacy and broaden its applicability. In conclusion, this study pioneers the investigation of BEO as a neuroprotective agent against heavy metal toxicity, marking an important step toward the development of natural remedies for neurodegenerative conditions, among which is AD. BEO demonstrated antibacterial activity further supports its potential as a multifaceted natural product. Although the results obtained in this study support the therapeutic potential of BEO in the context of neurodegenerative diseases, further studies are still necessary to ascertain its effectiveness in humans.

## Figures and Tables

**Table 1:** Chemical composition of BEO.

No	Compound ^a^	Class ^b^	RI ^c^	RI Lit. ^d^	Area % ± SD ^e^	ID ^f^
1	*α*-thujene	MH	924	924	0.22 ± 0.01	Std,RI,MS
2	*α*-pinene	MH	930	932	1.08 ± 0.00	Std,RI,MS
3	Sabinene	MH	970	969	0.77 ± 0.02	Std,RI,MS
4	*β*-pinene	MH	973	974	5.76 ± 0.04	Std,RI,MS
5	Myrcene	MH	989	988	0.69 ± 0.02	Std,RI,MS
6	*α*-terpinene	MH	1014	1014	0.05 ± 0.00	Std,RI,MS
7	*p*-cymene	MH	1022	1020	0.76 ± 0.00	Std,RI,MS
8	Limonene	MH	1026	1024	38.24 ± 0.32	Std,RI,MS
9	*γ*-terpinene	MH	1056	1054	6.58 ± 0.07	Std,RI,MS
10	Terpinolene	MH	1085	1086	0.15 ± 0.01	Std,RI,MS
11	Linalool	MO	1098	1095	14.45 ±0.09	Std,RI,MS
12	*α*-terpineol	MO	1187	1186	0.02 ± 0.00	Std,RI,MS
13	linalool acetate	MO	1256	1254	30.50 ± 0.16	RI,MS
14	neryl acetate	MO	1364	1359	0.17 ± 0.02	RI,MS
15	geranyl acetate	MO	1383	1379	0.16 ± 0.02	RI,MS
16	*α*-(*E*)-bergamotene	SH	1433	1432	0.07 ± 0.01	RI,MS
17	(*Z*)-*α*-bisabolene	SH	1507	1506	0.10 ± 0.02	RI,MS
	Total identified				99.77 ± 0.03	
	Monoterpene hydrocarbons				54.31 ± 0.37	
	Oxygenated monoterpenes				45.29 ± 0.10	
	Sesquiterpene hydrocarbons				0.17 ± 0.02	

^a^ Components are ordered according to their elution from the HP-5MS column. ^b^ Class: monoterpene hydrocarbons (MH), oxygenated monotepenes (MO), and sesquiterpene hydrocarbons (SH). ^c^ Linear retention index calculated according to the Van den Dool and Kratz formula. ^d^ Retention index taken from Adams. ^e^ Relative percentage values are mean of two independent analyses; SD, standard deviation. ^f^ Identification methods: Std, comparison with available analytical standard; RI, coherence of the calculated RI with those stored in the ADAMS (2007) [34] and NIST 17 (2017) [35] libraries; and MS, mass spectrum matching with respect to ADAMS, FFNSC (2012) [36], and NIST 17 MS [35] libraries.

**Table 2:** Antibacterial activity of BEO (MIC, % *v*/*v*) and levofloxacin (MIC, μg/mL).

MIC								
	Microorganisms							
	Gram-Positive				Gram-Negative			
	*E. faecalis* ATCC 29212	*S. aureus* ATCC 29213	*S. aureus* ATCC 25923	*S. aureus* ATCC 43300	*E. coli* ATCC 25922	*E. coli* ATCC 35218	*K. pneumoniae* ATCC 13883	*K. pneumoniae* ATCC 70063
BEO	2	0.5	0.5	1	1	2	4	4
Levofloxacin	2	0.5	0.5	0.5	0.06	0.12	8	8

## Data Availability

Data are contained within the article.
